# Mechanical performance and color stability of 3D-printed ceramic-filled resin following various bleaching regimens: an in vitro study

**DOI:** 10.1186/s12903-026-09400-5

**Published:** 2026-08-05

**Authors:** Tamer M. Hamdy

**Affiliations:** Restorative and Dental Materials Department, Oral and Dental Research Institute, National Research Centre (NRC), Giza, Dokki 12622 Egypt

**Keywords:** 3D-Printed Ceramic-Filled Resin, Dental bleaching, Mechanical properties, Surface properties, Color change

## Abstract

**Background:**

Recent material science advancements enabled a 3D-printable ceramic-filled resin for restorations. Additive manufacturing produces crowns, veneers, and mock-ups. Office and home bleaching agents contact teeth and restoration surfaces during application, potentially altering material properties.

**Objectives:**

The aim of the study is to assess the compressive strength, flexural strength, surface microhardness, surface roughness, and color changes of the additive 3D-printed ceramic-filled resin before and after exposure to home and in-office tooth bleaching.

**Methods:**

A total of 150 specimens were equally distributed into control (non-bleaching), home-bleaching (16% carbamide peroxide), and in-office bleaching (40% hydrogen peroxide) groups (*n* = 50/group). Compressive strength was evaluated using a universal testing machine, flexural strength by a four-point bending test, surface microhardness using a Vickers hardness tester, surface roughness using a 3D optical profilometer, and color changes using a spectrophotometer based on the CIEDE2000 (ΔE_00_) system. Statistical analysis was performed using one-way ANOVA, Tukey post-hoc tests, and independent t-tests (α = 0.05).

**Results:**

Both home bleaching (16% carbamide peroxide) and in-office bleaching (40% hydrogen peroxide) significantly reduced the compressive strength, flexural strength, and surface microhardness of the 3D-printed ceramic-filled resin while significantly increasing surface roughness (*P* ≤ *0.05*). The in-office bleaching group exhibited significantly greater reductions in mechanical properties and higher surface roughness values than the home-bleaching group. Although bleaching produced statistically significant color changes, all ΔE_00_ values remained below the clinically acceptable threshold.

**Conclusions:**

Home bleaching (16% carbamide peroxide) and in-office bleaching (40% hydrogen peroxide) reduced compressive and flexural strength, microhardness, and increased roughness of 3D-printed crowns. In-office bleaching showed greater effects, while color changes remained clinically acceptable.

**Supplementary Information:**

The online version contains supplementary material available at 10.1186/s12903-026-09400-5.

## Background

3D printing, also known as additive manufacturing, is a technique that produces objects by adding material layer by layer according to a digital model. In dentistry, Additive Manufacturing (AM) has attracted great interest, providing novel ways of producing a variety of components such as dental implants, crowns and bridges, indirect restorations, removable appliances, orthodontic devices, surgical guides, and bioprinting for regenerative dentistry. The applications of 3D printing are in continuous progress, providing great possibilities for the future of dental practice [1, 2].

Since there is an increasing tendency towards the widespread availability and proliferation of the 3D printing industry, various materials for the production of permanent fixed dentures have been presented [2]. Permanent crown resin is an emerging specialized tooth-colored, ceramic-filled resin material, specially designed for 3D-printed permanent indirect single-unit restorations, including crowns, inlays, onlays, and veneers. The workflow commonly employs a desktop stereolithography (SLA) printer, along with a matching build platform and post-processing (washing, curing, polishing), to fabricate the final restoration [3]. Permanent crown resin could be a solid choice for creating permanent dental restorations. It offers clinical benefits as high polishability and a smooth finish, which ensures a high aesthetic match. It also gives high mechanical strength due to the incorporation of ceramic fillers [4]. Moreover, the resinous component imparts suitable shock-absorbent properties. Additionally, the digital workflow delivers a high degree of accuracy, confirming an excellent marginal fit. Consequently, it is considered a respectable selection for single crowns, especially over dental implants, and in small bridges [4, 5].

3D-printed ceramic-filled resin restorations and teeth whitening have grown in popularity as aesthetic dentistry treatments due to patient demand for a perfect smile and improved awareness of aesthetics [6]. Tooth whitening has become extremely popular in the past decade owing to the increased accessibility of both at-home and in-office bleaching. Bleaching is a traditional, minimally invasive, conservative, uncomplicated, esthetically pleasing, and affordable technique that can be used to eliminate teeth discoloration and achieve a more attractive smile by removing tooth stains [7].

Most contemporary bleaching systems typically contain either hydrogen peroxide (HP) or carbamide peroxide (CP) [8]. Carbamide peroxide, when in contact with water, breaks down to hydrogen peroxide and urea molecules, whereby the hydrogen peroxide is the main whitening component. While home bleaching products on the market typically use between 3 to 20% carbamide peroxide, professional in-office bleaching systems utilize concentrations of hydrogen peroxide that range from 25%−40%, providing a quicker and deeper whitening result under a professional's direction [9].

Quality and longevity of indirect aesthetic restorations are significantly influenced by the mechanical properties and optical features of the restorative dental materials, such as compressive strength, flexural strength, surface microhardness, surface roughness, and color stability [10, 11].

Compressive and flexural strengths are significant to avoid restoration failure and to achieve durability of these materials, as the higher their compressive strength values, the more resistant restorations are against loads [12]. Higher flexural strength is essential for producing restoration in thin sections without the potential to bend under masticatory stresses and for long-term durability of the restoration [13, 14]. Furthermore, surface mechanical properties such as surface microhardness and surface roughness are beneficial as they provide resistance to scratching and provide a smooth surface, permitting restoration of surface integrity, subsequent optimal light reflection, and inhibition of plaque accumulation [15–17]. Additionally, color stability is a critical consideration, as it directly impacts the appearance of restorations over time [18].

While the effectiveness of dental bleaching for enhancing tooth color is well-demonstrated, there are discrepancies in its effects on restorative materials based on previous studies [19, 20].

In clinical tooth-whitening procedures, bleaching agents are inevitably exposed to existing restorations over extended amounts of time. It is therefore relevant to test restorative materials in conditions, which replicate home- and in-office bleaching protocols. In the present study, bleaching regimens were based on commonly accepted clinical protocols to closely simulate the cumulative exposure that restorations might receive after repeated individual whitening treatments.

Although several studies have investigated the influence of bleaching agents on conventional composite resins, CAD/CAM resin blocks, and ceramic materials, the available evidence regarding additively manufactured ceramic-filled permanent crown resins remains scarce and inconclusive [3]. Due to differences in material composition, polymerization mechanisms, filler characteristics, and post-curing protocols, findings obtained from conventional restorative materials cannot be directly extrapolated to 3D-printed ceramic-filled resins. Therefore, further investigation is required to determine whether bleaching procedures may adversely affect the mechanical, surface, and optical properties of these emerging restorative materials [21–23]. Therefore, this study aims to evaluate the impact of home and in-office bleaching on the compressive strength, flexural strength, surface microhardness, surface roughness, and color changes. This study seeks to understand how dental bleaching may influence the performance of 3D-printed ceramic-filled resin. The null hypothesis was that different bleaching protocols would not significantly affect the mechanical, surface, or optical properties of the investigated 3D-printed ceramic-filled resin.

## Methods

In this in vitro study, a tooth-colored, ceramic-filled resin designed for SLA 3D printing of permanent single crowns, inlays, onlays, and veneers (3D-printed permanent crown; Formlabs, Berlin, Germany) was used to create a 3D-printed ceramic-filled resin specimens. The dental bleaching agents used were as follows: 40% HP Office bleaching material (Opalescence Boost; Ultradent Products, Inc., South Jordan, UT, USA), and 16% CP home bleach (Opalescence PF; Ultradent Products, Inc., South Jordan, UT, USA) were applied. The compositions for the employed materials are shown in Table 1.

**Table 1:** The compositions for the employed materials

Commercial name	Category	Compositions	Batch Number	Manufacturer
3D-printed permanent crown	3D-printed ceramic-filled resin	Polymer matrix: Methacrylate-based Monomers/Oligomers of Esterification products of 4,4'-isopropylidenediphenol, ethoxylated and 2-methylprop-2-enoic acid Ceramic Micro- and Nanofillers: Silanized dental glass and pyrogenic silica (silica nanoparticles) (particle size 0.7 μm): 30–50 wt % Photoinitiators: Diphenyl (2,4,6-trimethylbenzoyl) phosphine oxide and methyl benzoylformate	FLPCA204	Formlabs Inc., Somerville, MA., USA
Opalescence PF	Teeth home whitening systems (Peroxide-based bleaching agent)	Carbamide peroxide 16%, potassium nitrate, sodium fluoride, glycerin, polyethylene glycol, and/or polyacrylic acid, water, sodium hydroxide, xylitol	BTW4K	Ultradent Products, Inc., South Jordan, UT, USA
Opalescence Boost	in-office professional teeth whitening system (High-concentration hydrogen peroxide)	Hydrogen peroxide 40%, 3% potassium nitrate, 1.1% sodium fluoride	BPMW6	Ultradent Products, Inc., South Jordan, UT, USA

### Sample grouping

The sample size calculation was determined by G*Power software version 3.1.9.7 (Heinrich Heine University Düsseldorf, Düsseldorf, Germany) for a power of 0.85 and α = 0.05 with a medium effect size of 0.5 according to a former studies [24–26]. The samples were equally divided into 3 main groups; control (non-bleaching) groups, the in-office bleaching groups and the at-home bleaching groups, Total sample size (n) was predicted as 150 samples (50 samples per group and 10 samples per subgroup). The study design involved independent control, home-bleaching, and in-office bleaching groups. Therefore, all evaluated properties were measured on separate specimens within each group, and comparisons were performed against the non-bleached control group.

### Sample fabrication

Samples were fabricated using a VAT photopolymerization-based 3D printing system; different specimen geometries were fabricated according to the requirements of each test. Cylindrical specimens (3 mm diameter × 6 mm height) were prepared for compressive strength testing, rectangular bar specimens (25 mm × 2 mm × 2 mm) were prepared for flexural strength testing according to ISO 4049:2019 [27], and disc-shaped specimens were prepared for surface microhardness, surface roughness, and color measurements.

SelfCAD software (CrossBrowser 3D LLC, NY, USA) was utilized for obtaining the design of each specimen in STL file format. Each of the specimens' designs was obtained using SelfCAD software (CrossBrowser 3D LLC, NY, USA) in STL files. This software provides import and tuning of the 3D model, as well as the ability to specify printing parameters such as layer thickness, print velocity, and curing time. The 3D printer subsequently loaded the print file to initiate printing [28, 29].

**Fig. 1:**
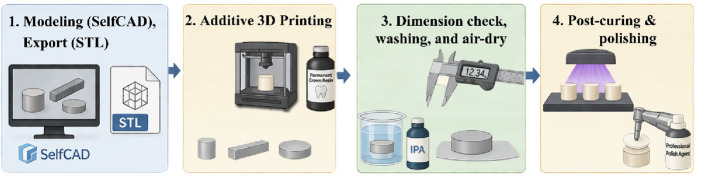
Diagrammatic flowchart representing sample fabrication steps

The models were then printed using 3D-printed permanent Crown Resin material (Formlabs Inc., Somerville, MA., USA) by using a high-resolution SLA 3D printer (Formlabs Inc., Somerville, MA., USA). with a 405 nm light source and a layer thickness of 50 µm as recommended by the printer manufacturer. The width, thickness, and length of all samples were verified by measuring their dimensions using a high-precision digital caliper (Digital Vernier Caliper, Mitutoyo, Japan) with an accuracy of 0.02 mm. The samples were rinsed in isopropyl alcohol for 3 min to clean any uncured resin on the surface. The specimens were subsequently allowed to air-dry before being subjected to two-step post-curing under a post-curing unit (UV-01 Thermostatic, Shenzhen PioCreat 3D Technology Co., Ltd, Shenzhen, China). After post-curing, all specimens were finished using sequential silicon carbide abrasive papers under water cooling and subsequently polished using (Renfert GmbH, Hilzingen, Germany) according to the manufacturer's instructions. The polishing compound was applied using a soft polishing wheel at low speed for a standardized duration to obtain a smooth and uniform surface before testing [28, 29]. Flowchart of the sample fabrication was represented in Fig. 1.

### Bleaching procedure

The selected bleaching regimens were designed to simulate commonly used clinical whitening protocols. The at-home protocol reflected the daily use of 16% carbamide peroxide trays, whereas the in-office protocol followed the manufacturer's recommendations for professional application of 40% hydrogen peroxide. The in-office bleaching protocol was established to closely imitate a single clinical whitening session determined by the manufacturer application protocol.

The 6-h daily application period for 16% carbamide peroxide was selected to simulate a clinically relevant overnight home-whitening protocol commonly recommended by the manufacturer and reported in previous studies. For the in-office bleaching procedure, Opalescence Boost was mixed immediately before application according to the manufacturer's instructions. The bleaching gel was periodically agitated using a microbrush every 5 min to ensure uniform contact with the specimen surface throughout the treatment period.

The specimens were categorized in 3 major groups: control, home bleaching (16% CP), and in-office bleaching (40% HP) (*n* = 50 for each). Each of them was divided into 5 sub-groups based on individual studied test (*n* = 10). The control group specimens were not bleached and were stored in distilled water for 14 days before the bleaching of other groups was carried out.

For the at-home bleaching groups, 16% CP gel was applied to the polished sample surface consistently using a manufacturer's syringe. The gel was evenly spread on sample surfaces with the help of an applicator. The CP was left on the samples at room temperature for 6 h per day. We then washed the gel away with deionized water until completely cleared from all surfaces. The discs were then immersed in distilled water for 18 h. We performed the above treatment for 14 days [25, 30].

**Fig. 2:**
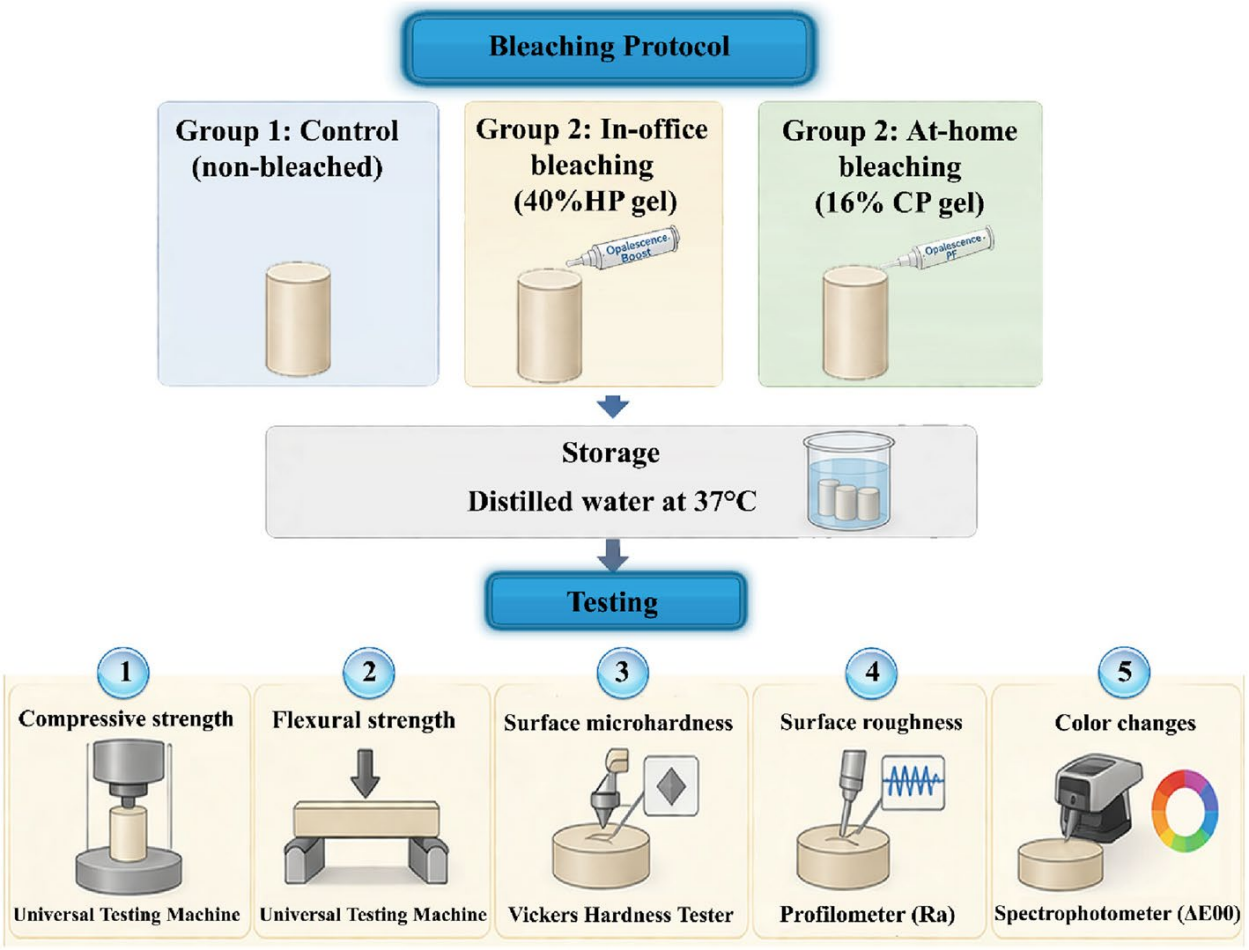
Diagrammatic flowchart representing the bleaching protocol and testing

For the in-office bleaching groups, uniform layer of 40% HP bleaching gel to the polished surfaces of the samples was applied using an applicator. The HP was applied on the specimens for 20 s and activated with a micro brush every five minutes, as recommended by the manufacturer. The specimen surfaces were washed with water for one minute and stored in distilled water at 37 °C After 20 min each time this cycle was repeated twice. Then the slices were stored in distilled water before testing [25, 30]. During the bleaching procedure, a standardized layer of bleaching gel was placed to completely cover the exposed surface of the specimen. Care was taken to maintain uniform gel distribution among all specimens during each application period. Following completion of the bleaching protocols, all specimens were rinsed thoroughly and subjected to the respective mechanical, surface, and color analyses immediately after the final storage period. The bleaching protocol and testing was illustrated in Fig. 2.

### Compressive strength analysis

A universal testing machine (Shimadzu Autograph AG–X Plus, Kyoto, Japan) was utilized to test a cylindrical specimens of 3 mm in diameter and 6 mm in height [27]. After the polymerization, specimens were taken out from the molds and stored in an incubator (CBM S.r.l. Medical Equipment: 2431/V; Cremona, Italy) for 24 ± 2 h at 23 ± 2 °C and a relative humidity of 50 ± 10%, specimens then investigated by applying a vertical load of 5 kN at a rate of 2 mm/min. until failure, according to ISO 4049:2019 [27].

### Flexural strength analysis

Flexural strength test was performed according to ISO 4049:2019 [27] standard using the 4-point bending technique. Four-point bending method was chosen due to its application within a large area of uniform tensile stress between the loading points and a smaller localized stresses concentration, enabling for better assessment of material properties of heterogeneous resin-based materials [31]. Rectangular cross-section samples (25 mm × 2 mm × 2 mm) were then fabricated [32]. These samples were then evaluated in a universal testing machine (Model 3345; Instron Industrial Products, Norwood, MA, USA). The load was placed at the center of each sample with a crosshead speed of 5 mm/min to fracture. The bending strengths of the specimens were calculated using the ISO 4049 standards [27].

### Vickers microhardness analysis

Vickers hardness test was chosen, since it is one of the methods most commonly used to check surface mechanical properties of dental resin materials and is a trustworthy standard for quantifying resistance to localized deformation [33]. Each specimen was examined before and after each type of bleaching to investigate surface microhardness using Vickers hardness tester (Shimadzu, HMVG31ST microhardness, Kyoto, Japan). The indenter is retained at load 100 g for a period of 15 s. at a magnification of 20 ×. The average surface microhardness obtained from each measurement taken for five indents for each sample is determined [34].

### Surface roughness analysis

The surface roughness (Ra) was evaluated by a 3D optical profilometry surface analyzer system (Amtast USA Inc., AMT211, Lakeland, FL, USA). Data were collected according to ISO 4287 specifications with a cutoff of 0.80 mm, resolution of 0.0001 mm (8-mm range), speed of 0.5 mm/s, total length was set to measure at least 4 mm in all cases. For each specimen, the average of the 3 measurements along both x- and y-axes was calculated and considered as a result [35].

### Color changes measurement

Color was recorded before application of each type of bleaching agent as base point value (T0). At the end of each bleaching process, the final (Tf) color values were measured for each specimen using extra-oral spectrophotometer (Cary 5000, Agilent Technologies, California, USA). The color difference (ΔE_00_) was calculated using the CIEDE2000 from the dip (Commission International de l’Eclariage) Lab* system against a black background under an illuminant D65[36]. The calculation based on the following formula [36–38]:

$${\Delta \mathrm{E}}_{00}=\sqrt{{\left(\frac{\Delta \mathrm{L}^{\prime}}{{\mathrm{k}}_{\mathrm{L}}+{\mathrm{S}}_{\mathrm{L}}}\right)}^{2}+{\left(\frac{\Delta \mathrm{C}^{\prime}}{{\mathrm{k}}_{\mathrm{C}}+{\mathrm{S}}_{\mathrm{C}}}\right)}^{2}+{\left(\frac{\Delta \mathrm{H}^{\prime}}{{\mathrm{k}}_{\mathrm{H}}+{\mathrm{S}}_{\mathrm{H}}}\right)}+\left({\mathrm{R}}_{\mathrm{T}}\left(\frac{\Delta \mathrm{C}^{\prime}}{{\mathrm{k}}_{\mathrm{C}}+{\mathrm{S}}_{\mathrm{C}}}\right) \left(\frac{\Delta \mathrm{H}^{\prime}}{{\mathrm{k}}_{\mathrm{H}}+{\mathrm{S}}_{\mathrm{H}}}\right)\right)}$$

where ΔL = difference in lightness, ΔC = difference in chroma, ΔH = difference in hue and the kL, SL, kC, SC, KH & SH are coefficient of constant.

The calculated ∆E_00_ values were compared to perceptibility threshold at which the first visual detectable color difference can be observed at (0.8) and the acceptability threshold which represents the starting point of unacceptable color change (∆E_00_ = 1.8) [39].

### Statistical analysis

Statistical study was performed using the Statistically Program for Social Science (SPSS) 16.0 software package program (IBM-SPSS version 27.0, NY, USA). The normality of the data was also confirmed by a Shapiro–Wilk test. One-way ANOVA and Tukey's post-hoc tests were used to statistically analyze the results of compressive and flexural strengths as well as microhardness and surface roughness between the control, home-, and office-bleached groups. While an independent sample t-test was used to compare the color changes after home- and office-bleaching. The level of significance was set as *P* ≤ *0.05*. In the color changes test, each specimen was measured at baseline (T0) and final (Tf) points to calculate the color difference (ΔE_00_) according to CIEDE2000. The obtained ΔE_00_ values were calculated as the outcome variable for statistical analysis. Because ΔE_00_ values from the home-bleaching and office-bleaching groups were obtained from independent specimens, comparisons were performed using an independent-samples t-test.

## Results

**Fig. 3:**
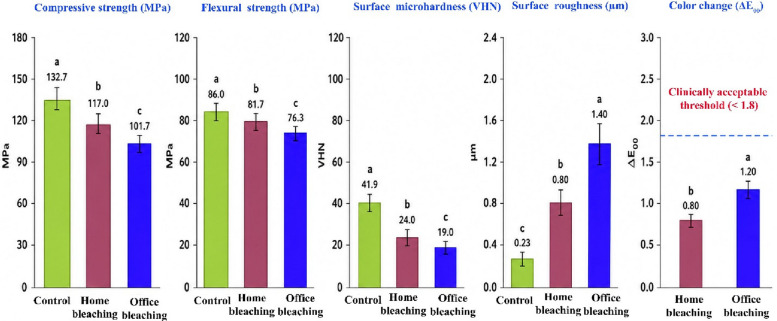
Bar chart summarizing the mean and standard deviation values of the compressive strength, flexural strength, surface microhardness, surface roughness, and color change of the investigated specimens. Different lowercase letters indicate statistically significant differences among groups (*P* < 0.05)

A summary of all test results was shown in Fig. 3. The results of the current study showed that there was a significant difference in the compressive strength between the three groups (*P* = *0.0001*). The control group displayed the highest compressive strength (132.7 MPa), followed by the home-bleached group (117 MPa), while the office-bleached group displayed the least compressive strength (101.7 MPa), as shown in Table 2.

**Table 2:** The mean and standard deviation values of the compressive strength

	Compressive strength (MPa) ± SD
Control	132.7^a^ ± 2.5
Home bleaching	117^b^ ± 1
Office bleaching	101.7^c^ ± 1.5
*P* value	0.0001*

^*^denotes a significant difference (*P* < *0.05*) for different small letters in the same column

There was a significant difference in the flexural strength between the three groups (*P* = *0.01*). The control group displayed the highest flexural strength (86 MPa), followed by the home-bleached group (81.7 MPa), while the office-bleached group displayed the least flexural strength (76.3 MPa), as represented in Table 3.

**Table 3:** The mean and standard deviation values of the flexural strength

	Flexure strength (MPa) ± SD
Control	86^a^ ± 1
Home bleaching	81.7^b^ ± 1.1
Office bleaching	76.3^c^ ± 1.5
*P* value	0.01

^*^denotes a significant difference (*P* < *0.05*) for different small letters in the same column

There was a significant difference in the surface microhardness between the three groups (*P* = *0.0001*). The control group displayed the highest surface microhardness (41.9 VHN), followed by the home-bleached group (24 VHN), while the office-bleached group displayed the least surface microhardness (19 VHN), as presented in Table 4.

**Table 4:** The mean and standard deviation values of the surface microhardness

	Microhardness ± SD (VHN)
Control	41.9^a^ ± 1.1
Home bleaching	24^b^ ± 0.1
Office bleaching	19^c^ ± 0.2
*P* value	0.0001*

^*^denotes a significant difference (*P* < *0.05*) for different small letters in the same column

There was a significant difference in the surface roughness (Ra) between the three groups (*P* = *0.0001*). The control group displayed the least surface roughness (0.23 µm), followed by the home-bleached group (0.8 µm), while the office-bleached group displayed the highest surface roughness (1.4 µm), as demonstrated in Table 5.

**Table 5:** The mean and standard deviation values of the surface Roughness (Ra)

	Surface Roughness** (**µm) ± SD
Control	0.23^a^ ± 0.04
Home bleaching	0.8^b^ ± 0.04
Office bleaching	1.4^c^ ± 0.08
*P* value	0.0001*

^*^denotes a significant difference (*P* < 0.05) for different small letters in the same column

There was a significant difference in the color changes (ΔE_00_) between the home- and office-bleached groups (*P* = *0.0001*) relative to the baseline color measurements (T0) obtained for each specimen before bleaching. The home-bleached group displayed a lower color change (ΔE_00_ = 0.8) than the office-bleached group, which displayed the highest color change (ΔE_00_ = 1.2), as shown in Table 6.

**Table 6:** The mean and standard deviation values of the color changes (ΔE_00_)

	Color changes (ΔE_00_) ± SD
Home bleaching	0.8^a^ ± 0.01
Office bleaching	1.2^b^ ± 0.01
*P* value	0.0001*

^*^denotes a significant difference (*P* < *0.05*) for different small letters in the same column

## Discussion

Additive manufacturing has emerged as a promising technology in restorative dentistry, offering efficient fabrication of highly esthetic restorations with reduced material waste and streamlined digital workflows [40]. Among these materials, 3D-printed ceramic-filled resins have gained increasing attention because of their favorable mechanical properties, esthetic potential, and clinical versatility. Given the growing popularity of both 3D-printed restorations and tooth-whitening procedures, understanding the influence of bleaching agents on the performance of these materials is clinically important. Therefore, the present study evaluated the effects of home and in-office bleaching on the mechanical, surface, and optical properties of a 3D-printed ceramic-filled resin [41].

Tooth bleaching is a widely used and minimally invasive treatment for improving dental esthetics. However, because bleaching agents inevitably contact existing restorations during treatment, concerns have been raised regarding their potential effects on the structural integrity, surface characteristics, and color stability of restorative materials [42, 43]. Since these properties are critical determinants of the longevity and clinical performance of resin-based restorations, the present study investigated the effects of home and in-office bleaching protocols on the compressive strength, flexural strength, surface microhardness, surface roughness, and color stability of a 3D-printed ceramic-filled resin [18, 44, 45]. Therefore, the aim of this study was to evaluate the effect of home in-office tooth bleaching on the compressive strength, flexural strength, surface microhardness, surface roughness, and color changes of the additive 3D-printed ceramic-filled resin.

The four-point bending test is an advantage of the present study, since the stress is mostly uniform in the middle span compared to the three-point bend. Thus, it better evaluates brittle or heterogeneous materials such as ceramic-filled resin composites and also allows for a more precise comparison of material performances amongst different studies [31]. The Vickers method was used to evaluate the surface microhardness, a common mechanical property that indicates how well resin-based materials can withstand abrasion. Changes in microhardness may reflect alterations within the polymer matrix and can serve as an indirect indicator of material degradation following bleaching exposure [33]. The present study simulated a single in-office bleaching session rather than multiple treatment appointments. Therefore, the observed changes represent the immediate effects of one professional bleaching procedure. Repeated bleaching sessions, which may be performed clinically depending on patient needs, could potentially result in greater alterations to the evaluated properties and should be investigated in future studies.

The null hypothesis was rejected, as it was observed that the home and in-office bleaching produced a significant effect on the compressive strength, flexural strength, surface microhardness, roughness, and color alterations of 3D-printed ceramic-filled resin. The bleached groups of the 3D-printed ceramic-filled resin demonstrated a significant decrease in compressive strength, flexural strength, and surface microhardness with an increased surface roughness. The in-office bleaching has more pronounced effects than that of home bleaching.

The reduction in compressive and flexural strength observed after bleaching may be associated with oxidative degradation processes previously reported for resin-based materials. Peroxide-derived free radicals have been suggested to affect the polymer matrix and resin-filler interface, potentially contributing to mechanical deterioration [28]. However, because no microscopic or chemical analyses were performed in the present study, these mechanisms remain speculative and should be interpreted with caution.

The decrease in surface microhardness may be related to elimination and alterations in the organic matrix induced by peroxide exposure, as suggested in previous investigations. [46]. Nevertheless, the present study did not include chemical characterization methods capable of confirming this mechanism. The increase in surface roughness may be associated with degradation phenomena occurring at the resin-filler interface [24]. However, without SEM or other surface characterization techniques, the exact nature of these surface alterations cannot be confirmed.

The observed reductions in compressive and flexural strength may not directly correlate to immediate restoration failure, but repeated applications of bleaching agents may contribute to cumulative degradation and decrease longevity over time [47]. Likewise, the surface roughness evolution observed after bleaching could favor plaque retention, greater bacterial adhesion, staining susceptibility, and loss of gloss [48]. These effects were more pronounced in the case of the in-office bleaching group, indicating that high peroxide concentrations seem to have a greater effect on the retention of existing restorations [49, 50].

The peroxides in bleaching agents for teeth may cause oxidative cleavage of polymer chains. This is the point not reacted double bonds are a weak point in the polymer. Furthermore, free radicals generated by these peroxides may influence the resin-filler interface and contribute to debonding [7]. The obvious effect of in-office bleaching on the mechanical properties than home-bleaching may be due to using high concentrations of hydrogen peroxide (40%) and the subsequent more intense interaction with the organic matrix and filler-resin interface [24]. Clinically, higher surface roughness can facilitate plaque accumulation, bacterial adhesion, and staining while decreasing surface gloss that affects esthetic attributes. Consequently, the higher roughness noticed after in-office bleaching may negatively impair the longevity of restorations [51].

On the other hand, the color changes (ΔE_00_) among all the bleached groups were below the clinically acceptable threshold (< 1.8) [52]. In clinical practice, the acceptability of color change is categorized based on specific perceptibility and clinical thresholds using the CIEDE2000 (ΔE_00_) formula. A value of ΔE_00_ represents the limit of visual perceptibility, where any color change is considered indistinguishable to the human eye and therefore clinically excellent. When the color change falls between 0.8 and 1.8, it reaches the threshold of clinical acceptability; while the shift may be detectable by a trained clinician, it remains aesthetically pleasing and acceptable to the patient [52]. Conversely, a value of ΔE_00_ > 1.8 exceeds the clinical acceptability threshold, signifying a visually disharmonious result that is deemed clinically unacceptable in restorative dentistry [52].

The color stability of the 3D-printed ceramic-filled resin may be attributed to the 3D printing process, where the layer-by-layer additive process results in the production of a highly organized and densely cross-linked methacrylate polymer network. This dense network may limit the penetration of the bleaching agents [53]. Additionally, the post-curing procedure that is performed after the printing process enhances the degree of conversion and chemical resistance by reducing the chemical sites that bleaching agents could attack [54]. Moreover, a previous study conducted by *Raszewski *et al*.* (2026), demonstrated that the post-curing protocol significantly influences the long-term color stability of 3D-printed dental resins [30]. All ΔE_00_ values were statistically significant after bleaching, but never reached a clinically unacceptable level (ΔE00 < 1.8). Thus, the changes in color are not likely to reduce the esthetic quality of restorations in clinical practice.

The findings of the present study are consistent with those reported by *Atay *et al*.* (2025), who concluded that restorations fabricated from CAD/CAM-milled and adequately post-cured 3D-printed resins exhibit greater resistance to bleaching-induced alterations than conventional resin composites [48]. In contrast, *Alharbi *et al*.* (2021) reported that exposure to various immersion media resulted in substantial color changes in both 3D-printed and CAD/CAM-milled resin-based materials [55]. This discrepancy may be attributed to differences in the aging protocols, immersion media, material composition, and experimental conditions employed across the studies.

The present findings are also consistent with recent investigations evaluating permanent printable restorative resins, which were conducted by *Sharshar *et al*.* (2026), who reported increases in surface roughness of the tested additively manufactured 3D-printed resin materials compared to CAD/CAM resin-based restorative materials after exposure to peroxide-based in-office bleaching [56].

This finding coincides with that of *Duarte *et al. (2024), who reported that the filler content of commercially available 3D printing resin-based composites is significantly lower than that of CAD-CAM resin-based composites; such differences may be attributed to variations in ceramic filler loading, resin matrix composition, printing technology, and post-curing protocols. They also determined that 3D printed resin-based composites tend to be more appropriate for provisional applications [57].

Furthermore, *Dascanio *et al*.* (2026) demonstrated that 3D-printed resin materials exhibit acceptable short-term clinical performance, indicating their suitability for provisional indirect restorations. Nevertheless, evidence regarding their long-term durability remains limited. In contrast, CAD/CAM milled restorative materials have consistently shown superior mechanical properties and optical stability over time. These findings underscore the importance of further long-term clinical studies to validate the performance and longevity of permanent 3D-printed ceramic-filled restorative materials [58].

Several limitations should be considered when interpreting the findings of this study. As an in vitro investigation, it could not fully replicate the complex oral environment, including the effects of saliva, temperature fluctuations, biofilm formation, mechanical fatigue, and long-term aging. In addition, thermocycling and storage in artificial saliva were not performed. Furthermore, microscopic and chemical characterization techniques, such as SEM, FTIR, water sorption analysis, and degree-of-conversion measurements, were not included; therefore, the mechanisms underlying the observed changes could not be directly verified. Future studies incorporating these parameters are recommended to simulate clinical conditions better and provide a more comprehensive understanding of the effects of bleaching on 3D-printed ceramic-filled resin restorations. Although color changes were evaluated using the CIEDE2000 (ΔE_00_) formula, which is widely accepted for assessing color differences in dentistry, the Whiteness Index for Dentistry (WID) may provide additional information regarding whitening efficacy and perceptual whiteness changes. Future investigations may benefit from incorporating WID analyses for a more comprehensive color assessment.

## Conclusions

Home bleaching with 16% carbamide peroxide or in-office bleaching with 40% hydrogen peroxide can decrease compressive strength, flexural strength, and surface microhardness, as well as increase the surface roughness of 3D-printed permanent crowns. In-office bleaching was observed to have a more pronounced effect. However, color remained unaffected, and the observed changes were within clinically acceptable limits.

## Supplementary Information

Supplementary Material 1.

## Data Availability

The datasets generated during and/or analyzed during the current study are not publicly available due to institutional policy but are available from the corresponding author on reasonable request.

## Abbreviations

AM: Additive Manufacturing
SLA: Stereolithography
CP: Carbamide peroxide
HP: Hydrogen peroxide
ISO: International Standard Organization
SD: Standard deviation
MPa: Megapascal
VHN: Vickers Hardness Number
CAD/CAM: Computer-Aided Design and Computer-Aided Manufacturing
