# Supplementary material for: Mechanical performance and color stability of 3D-printed ceramic-filled resin following various bleaching regimens: an in vitro study
Source: BMC Oral Health. 2026 Aug 5;26:1374. doi: 10.1186/s12903-026-09400-5 (PMC13440038; doi:10.1186/s12903-026-09400-5)
Supplement: Supplementary file 1 [file 12903_2026_9400_MOESM1_ESM.docx]

**Figure 4:** diagrammatic flowchart of the sample grouping.
